# Morphological and genetic diversity of *Beta maritima* populations across Europe and North Africa: a comprehensive review

**DOI:** 10.3389/fpls.2025.1731515

**Published:** 2026-01-15

**Authors:** Lisa Bertram, Matthias Frisch

**Affiliations:** 1 Institute of Agronomy and Plant Breeding II, Justus Liebig University, Giessen, Germany; 2 KWS SAAT SE & Co. KGaA, Einbeck, Germany

**Keywords:** *Beta vulgaris* ssp. *maritima*, crop wild relatives, genetic diversity, genetic resources, sea beet

## Abstract

*Beta vulgaris* ssp. *maritima* (sea beet), the wild ancestor of cultivated beet, represents a key reservoir of adaptive genetic diversity for sugar beet breeding. This review synthesizes research on morphological and genetic variation of *Beta maritima* populations across Europe and North Africa to (1) summarize regional diversity patterns, (2) assess the correspondence between phenotypic traits and genetic structure, and (3) identify knowledge gaps. Morphological studies show wide variation in sea beet. Growth habits range from prostrate to erect. Coastal plants often have thicker leaves and prostrate forms while inland types are adapted for water efficiency. Traits like pigmentation, inflorescence, and root shape also differ, reflecting adaptation to local environments. Bolting and flowering occur early in Mediterranean populations but are delayed in northern regions. Genetic analyses further identify a distinct Atlantic/Mediterranean divide. Mediterranean populations exhibit greater genetic diversity, while Baltic populations show low diversity and high homogeneity, presumably due to recent establishment and founder effects. Comparative findings suggest phenotypic variation often exceeds genetic differentiation and is strongly influenced by environmental factors. This review identifies research gaps among sea beet populations in Mediterranean regions particularly along the southern and eastern coasts of Spain, Italy, Greece, Turkey, and the eastern Mediterranean. As the first comprehensive review focused solely on *Beta maritima in-situ* populations, this work delivers a full account of the regions, traits, and genetic patterns studied to date. It establishes a foundation for future research and is an indispensable resource for advancing breeding, conservation, and scientific understanding of this important wild relative.

## Introduction

**Figure 1:**
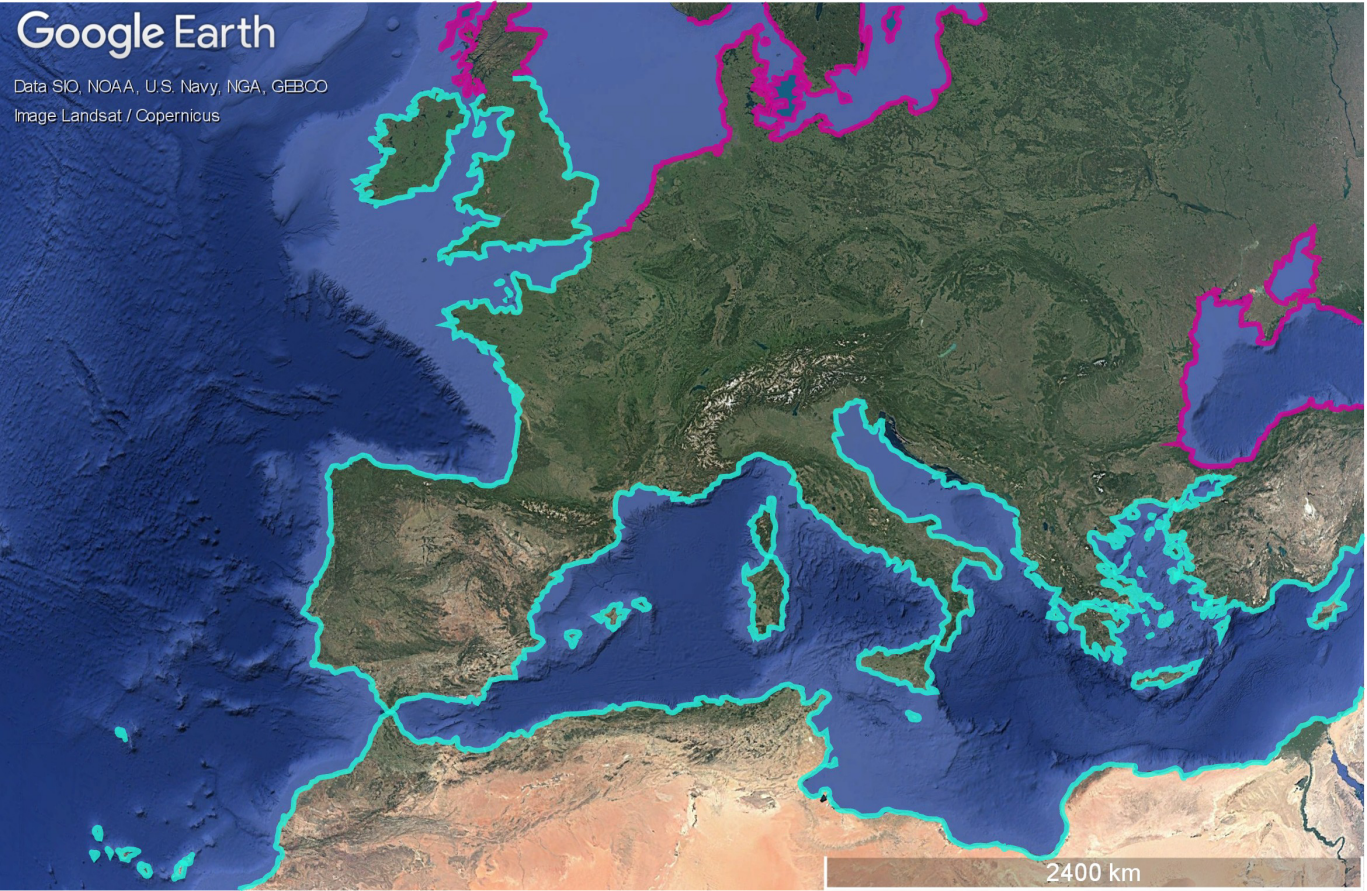
Map showing the general distribution of *Beta maritima* along the seashores within its main distribution area in Europe and Northern Africa (blue: frequent; purple: sparse). The map was generated using GoogleEarthPro. Adapted from Frese and Ford-Lloyd (2020).

*Beta maritima* (*Beta vulgaris* ssp. m*aritima* (L.) Arcang.; sea beet), the wild ancestor of cultivated beet, is the most widespread taxon within the genus *Beta* (Frese and Ford-Lloyd, 2020; **Figure 1**). Its origin is traced to the Mediterranean region (Romeiras et al., 2016). Its distribution covers nearly all Mediterranean coastal countries, several Atlantic islands, and much of the Atlantic coast of Europe (Frese and Ford-Lloyd, 2020; Veloso et al., 2021; Ben Mahmoud et al., 2025). Following the last glacial period, Be*ta maritima* expanded northward, establishing populations along the Atlantic and North Sea coasts (Doney et al., 1990; Boudry et al., 2002; Fievet et al., 2007; Monteiro et al., 2013). Some populations are large and well-established, while others are small and scattered, reflecting both historical dispersal and recent colonization events (Letschert, 1993; Driessen, 2003; Andersen et al., 2005; Frese and Ford-Lloyd, 2020).

The broad distribution of *Beta maritima* reflects its evolutionary success and its ecological adaptability. It thrives in highly variable and often harsh coastal habitats, including salt marshes, beaches, and inland ruderal sites (Doney et al., 1990; Stevanato et al., 2001). This environmental heterogeneity, combined with strong local selection pressures, drives high morphological variation within and among populations (Toll and Hendriksen, 1982; Letschert and Frese, 1993; Abdelhameed et al., 2024; Ben Mahmoud et al., 2025). Traits such as growth habit, leaf morphology, and bolting behavior are shaped by adaptation to salinity, drought, and temperature extremes (Letschert and Frese, 1993; El Manhaly et al., 1996; Ben Mahmoud et al., 2025). The species’ ability to colonize diverse habitats and maintain dynamic, *in-situ* populations preserves adaptive alleles that may be lost in *ex-situ* collections (Bohra et al., 2021).

Understanding the diversity of wild crop relatives like *Beta maritima* is essential for conservation and breeding efforts. Sea beet populations provide a reservoir of adaptive genetic variation, contributing valuable traits to sugar beet improvement (Frese et al., 1990; Panella et al., 2020). Over the past decades, numerous studies have documented the morphological variation among sea beet populations. In parallel to morphological investigations, advances in molecular genetics have enabled deeper insights into the population structure and genetic diversity of *Beta maritima*.

This review provides an overview of current research on the morphological and genetic diversity of *Beta maritima* populations across Europe and North Africa. It aims to (1) summarize regional patterns of morphological and genetic variation, (2) evaluate the relationship between observed phenotypic traits and underlying genetic structure, and (3) identify knowledge gaps and underexplored populations to support the utilization of *Beta maritima* as a genetic reservoir for sustainable sugar beet improvement.

## Morphological variation

Morphological diversity in *Beta maritima* has been extensively studied, with numerous investigations exploring how geographic and environmental factors shape variation within and among populations (**Table 1**). Across its native range, sea beet populations exhibit pronounced variability in growth habit, leaf morphology, inflorescence structure, pigmentation, and root traits (Toll and Hendriksen, 1982; Letschert and Frese, 1993; Stevanato et al., 2001; Abdelhameed et al., 2024; Ben Mahmoud et al., 2025).

**Table 1:** Summary of morphological diversity in *Beta maritima* populations examined across different studies.

Traits examined	Patterns of variation and key findings
Toll and Hendriksen, 1982 (Italy)	
	▪ High variability in growth habit, leaf size, inflorescence, seedball size, and pigmentation ▪ Variation in root tapering and side root number ▪ Greater variation between populations than within ▪ Morphology strongly linked to environment: ▪ Dry sites: small plants with thick leaves ▪ Open habitats: procumbent or prostrate growth
Doney et al., 1990 (England, Ireland, North Ireland, Wales)	
	▪ British Isles sea beets: few leaf hairs, heavy waxy cuticle, very green appearance ▪ Life cycle variation: annual and perennial forms observed; some populations highly uniform, others highly variable (older, established) ▪ Greater morphological differences with increasing distance and geographic barriers
Frese et al., 1990 (Portugal, Spain)	
	▪ Growth habit varied: erect types frequent on Iberian Peninsula ▪ Plant size highly variable; old populations produced large seed quantities ▪ Despite geographic barriers, little morphological differentiation - spatial separation does not seem to prevent gene flow ▪ Bay of Aerosa: exceptionally high morphological variability observed; may result from admixture of South and North Atlantic gene pools
Letschert and Frese, 1993 (Italy)	
*15 morphological characters:* pigmentation, leaf pubescence, bract shape, multigermicity, growth habit, flowering stage (2 periods), lamina (length, width, thickness), petiole (length, width), stem diameter, biomass (fresh weight), plant height	▪ High variation in lamina length, width, and thickness, petiole length and width, stem diameter, biomass, plant height, flowering (both periods) ▪ Tendency for decreasing leaf length and width from north to south ▪ No single trait reflected clear regional pattern ▪ Significant differences between adjacent populations; coastal populations showed large fluctuations ▪ Inland vs. maritime groups differed for some characters
El Manhaly et al., 1996 (Egypt)	
*18 morphological characters:* growth habit, leaf (erectness, hairiness, thickness), leaf blade (length, width, pigmentation), petiole (length, width), petiole color, hypocotyl pigmentation, external root color, main color flesh, root shape, flower stern pigmentation, male sterility, multigermicity, bolting tendency *9 evaluators for resistance:* curly top, Rhizoctonia, leaf spot, cyst nematode, root aphid, Rhizomania, virus yellows, root maggot, Aphanomyces	▪ Variation in growth habit, bolting behavior, leaf shape, roots ▪ Two main groups identified: ▪ Delta: prostrate, small thick leaves, no leaf hair ▪ Luxor/Fayum: segregating for leaf size, growth habit, root swelling, bolting, and red types ▪ Inland types had longer, narrower petioles and less succulent leaves than coastal types ▪ Bolting occurred very early, complicating disease resistance scoring ▪ Moderate curly top resistance found in Fayum/Luxor accessions; root aphid resistance noted in some populations
Bartsch and Schmidt, 1997 (Italy)	
*8 morphological characters:* bigermity, hypocotyl color, leaf (color, pubescence), pollen sterility, seed (cluster weight, emergence), annuality	▪ Differentiation into three types based on morphological characters ▪ Evidence of hybridization between wild beets and cultivars found (i.e. supported by bigermous individuals, low proportion of annual individuals [26.5%] among offspring)
Van Dijk et al., 1997 (Belgium, England, France, Guernsey, Jersey)	
vernalization requirements, sensitivity for vernalizing factors, flowering date	▪ Strong variation in flowering time is linked to latitude and vernalization requirement (mainly determined by a single gene *B/b* and quantitative trait loci) ▪ Southern populations: high frequency of *B* allele (no vernalization needed); northern populations: strong vernalization requirement ▪ High heritability (0.33) for flowering time → potential for evolutionary change.
Stevanato et al., 2001 (Italy)	
	▪ Significant morphological variability in leaf shape and size; deep green leaves, occasional red streaks ▪ Variability in the reproductive structure (seed stalks) ▪ Populations showed resistance to Cercospora and Rhizomania
Boudry et al., 2002 (Belgium, France, The Channel Islands)	
Vernalization requirements, flowering response to cold periods at young seedling stage	▪ Vernalization requirement increased with latitude; northern plants require more vernalization ▪ Young seedlings more difficult to vernalize than plants which already developed vegetative rosettes ▪ Penetrance of the annual habit in *Bb* genotypes was affected by both environmental and genetic factors
Ascarini et al., 2021 (Portugal)	
*11 morphological characters:* number of basel stems, plant height, inflorescence height, distance of the first branch from the basis, number of branches, leaf (length, width), petiole (length, width), stem diameter, average number of glomerulus per branch	▪ Morphological characterization showed a high quantitative variation among populations ▪ Plant height and inflorescence height parameters had the highest influence in the separation of populations ▪ Populations in less exposed sites tend to have bigger and more developed aerial parts; plants closer to the sea or under dry/saline conditions are more prostrate with smaller leaves
Abdelhameed et al., 2024 (Egypt)	
*35 morphological characters:* plant length, stalk (length, diameter), number of branches/stalk, branch length, lower & upper leaf lamina (length, width), lower & upper leaf petiole (length, width), inflorescence bract lamina (length, width), inflorescence bract petiole (length, width), lower & upper glomerule bract lamina (length, width), lower & upper glomerule bract petiole (length, width), lower & upper glomerule (length, width), lower & upper glomerule bract length/glomerule length, inflorescence/branch, number of glomerule/inflorescence, number of flowers/glomerule	▪ Notable morphological diversity observed within and among populations ▪ Significant differences in 17 of 35 traits; branch length and inflorescence traits showed highest variability ▪ Two groups identified: ▪ *var. glabra:* glabrous, erect, large basal leaves ▪ *var. pilosa:* hairy, prostrate, smaller basal leaves ▪ Soil parameters significantly influenced population morphological variability; especially strong correlation to soil organic carbon
Ben Mahmoud et al., 2025 (Tunisia)	
*23 morphological characters:* growth habit, stem (color, pigmentation, hairiness), leaf (color, pigmentation, curliness, hairiness, shape, blade length, blade width), petiole (color, length, width), cuticle thickness, bract (shape, thickness), inflorescence (color, height), multigermicity, flowering pattern between plants, glomerule diameter, 1000 seed weight	▪ Substantial morphological variability within and between populations ▪ Traits like glomerule diameter, seed weight, and inflorescence height showed high between-population variability ▪ Island populations: prostrate habit, red inflorescences ▪ Mainland populations: erect habit, hairy curly leaves ▪ Strong phenotypic plasticity; Adaptation to harsh conditions (salinity, heat, drought) evident, i.e. reducing leaf size allows plants to reduce water loss by evapotranspiration

The table lists the main traits analyzed, key findings, and principal patterns of variation observed within each study. Countries in brackets indicate the origin of the analyzed populations.

Morphological differentiation is strongly influenced by environmental factors. Traits such as plant size, leaf thickness, and growth form are strongly linked to habitat conditions. Plants in dry or exposed environments tend to be smaller with thicker leaves, while those in open, resource-rich habitats often develop more expansive, procumbent forms (Toll and Hendriksen, 1982; Abdelhameed et al., 2024; Ben Mahmoud et al., 2025). Exposure to stressors like high salinity, drought, or heat further drives adaptive changes, including reduced leaf size and increased cuticle thickness (Ben Mahmoud et al., 2025). Soil properties, especially organic carbon content, also significantly influence morphological variability (Abdelhameed et al., 2024).

A consistent pattern emerges when comparing inland and coastal populations. Inland types typically have longer, narrower petioles and less succulent leaves, whereas coastal populations display shorter, thicker leaves and a more prostrate growth habit (Letschert and Frese, 1993; El Manhaly et al., 1996). Coastal populations are also more likely to bolt and flower early, while inland types often show delayed generative development. These patterns reflect adaptation to contrasting environmental pressures. Coastal habitats favor compact, robust morphology, while inland environments select for traits that enhance water use efficiency and competitive ability (Letschert and Frese, 1993; El Manhaly et al., 1996).

The extent to which morphological variation correlates with geography is variable. Some studies report substantial diversity within short coastal stretches (Abdelhameed et al., 2024), while others find only minor divergence across broader regions (Letschert and Frese, 1993). In the British Isles, morphological variation increases with geographic distance and physical barriers, highlighting the influence of dispersal mechanisms and environmental heterogeneity (Doney et al., 1990).

A clear distinction is observed between Atlantic and Mediterranean populations. Mediterranean populations generally exhibit greater morphological and genetic diversity, with a wider range of growth forms and adaptive traits, while Atlantic populations, especially those in the Baltic and North Sea regions, are more uniform and often display traits associated with recent colonization and founder effects (Frese et al., 1990; Richards et al., 2014; Andersen et al., 2005). This regional contrast is also reflected in the frequency of key adaptive alleles, such as gene *B*, which controls vernalization requirement and flowering time. The *B* allele, which eliminates the need for vernalization and promotes early bolting, is frequent in Mediterranean populations but largely absent in northern populations, contributing to the observed differences in life history strategies (Van Dijk et al., 1997; Boudry et al., 2002).

Much of this diversity is attributable to phenotypic plasticity – *Beta maritima’s* ability to adapt its morphology in response to environmental variation (Ribeiro et al., 2016; Ascarini et al., 2021). Nevertheless, genetic control is also evident, as shown by the identification of major genes (e.g. gene *B*) and quantitative trait loci affecting flowering and growth (Van Dijk et al., 1997; Boudry et al., 2002). The interplay between plasticity and genetic differentiation complicates the interpretation of regional patterns and underscores the need for integrative approaches.

Beyond basic morphology, few studies also described variation in agronomically relevant traits such as disease resistance and stress tolerance. For example, resistance to important pathogens such as Cercospora leaf spot and Rhizomania has been documented in certain wild populations (Stevanato et al., 2001). Moderate resistance to curly top virus and root aphid has also been identified in populations from Egypt (El Manhaly et al., 1996). However, most findings are based on phenotypic screening rather than genetic confirmation. Nevertheless, these findings underscore the value of *Beta maritima* as a genetic resource for breeding programs, offering a reservoir of adaptive traits that can be harnessed to improve stress tolerance and disease resistance in cultivated beets.

## Genetic diversity

Following extensive research on morphological variation in wild sea beet, recent studies have increasingly focused on genetic diversity and population structure using molecular techniques (see **Table 2** for an overview of diversity measures across studies). These genetic analyses have uncovered both broad-scale patterns and distinct regional differences within *Beta maritima* populations. Importantly, genetic differentiation across Europe and North Africa reflects a complex, multi-layered process. Rather than being determined solely by geographic distance, population structure in *Beta maritima* populations is shaped by a combination of historical events, dispersal mechanisms, and environmental heterogeneity.

**Table 2:** Overview of diversity measures reported across studies. .

Study	Country												Number and type of genetic marker	N_A_	A_R_	N_P_	H'	H_O_	H_E_	F_IT_	F_ST_	F_IS_
	SE	DK	DE	IE	NL	GG	JE	FR	PT	ES	IT	MA										
Desplanque et al., 1999								X					5 RFLP & 1 microsatellite	3 – 26				0.508 – 0.796	0.609 – 0.900		0.020 – 0.204	0.085* – 0.318**
Driessen et al., 2001		X	X										75 RAPD-PCR fragment loci			8.1 – 27.0	0.046 – 0.144					
Driessen, 2003	X	X	X										78 RAPD-PCR fragment loci & 1 AFLP			6.4 – 34.6	0.038 – 0.182					
Andersen et al., 2005	X	X		X	X			X			X		8 SSR	1 – 8				0.040 – 1.000	0.040 – 0.810		(0.310***)	-0.800* – 0.530^NS^
Fievet et al., 2007						X	X	X					7 micro- & 4 minisatellites	3.85 – 7.14	3.350 – 5.580				0.410 – 0.600	0.086*** – 0.207*** (0.151***)	0.015^NS^ – 0.119*** (0.089***)	-0.012^NS^ – 0.100*** (0.068***)
Fénart et al., 2008								X					5 micro-, 4 minisatellites & 1 PCR-RFLP	1 – 6	1.000 – 5.410				0.000 – 0.880		0.147***	-0.441^NS^ – 0.439***
Leys et al., 2014								X	X	X		X	8 micro- & 4 minisatellite	3 – 56	3.650 – 12.043				0.418 – 0.831	0.132* – 0.223*	0.126* – 0.177*	0.007^NS^ – 0.087*
Richards et al., 2014								X	X				13 SSR	3 – 31	2.020 – 6.320			0.290 – 0.690	0.400 – 0.690		(0.140)	
Ribeiro et al., 2016									X				6 SSR	3 – 15	7.670 – 10.170			0.480 – 0.906	0.300 – 0.900		0.019 – 0.121 (0.052)	
Ascarini et al., 2021									X				8 SSR	22 – 38			0.856 – 1.381	0.400 – 0.650	0.575 – 0.787			-0.423 – 0.622 (0.186)
Veloso et al., 2021								X	X	X			6 SSR	7 – 25	2.333 – 10.167			0.044 – 0.783	0.074 – 0.789		0.000 – 0.277	-0.041 – 0.287
Bertram et al., 2025a		X		X				X					16,201 SNPs					0.099 – 0.167	0.430 – 0.780			

Countries from which populations were examined, SE, Sweden; DK, Denmark; DE, Germany; IE, Ireland; NL, The Netherlands; GG, Guernsey; JE, Jersey; FR, France; PT, Portugal; ES, Spain; IT, Italy; MA, Morocco. Diversity measures include N_A_, number of alleles per locus; A_R_, allelic richness; N_P_, polymorphic fragments (%); H′, Shannon Index; H_O_, observed heterozygosity; H_E_, expected heterozygosity; and F-statistics (F_IT_; F_ST_; F_IS_). Ranges of minimum and maximum values are shown; mean values are provided in brackets where available. Significance levels; if specified in the original studies: * = p < 0.05; ** = p < 0.01; *** = p < 0.001; NS, non-significant.

A key pattern of variation, the Atlantic–Mediterranean divide, originates from glacial history. Mediterranean regions acted as refugia during the last glacial period, preserving high allelic richness and heterozygosity. Northern populations on the other hand were recolonized more recently, resulting in reduced diversity and greater genetic uniformity within these populations (Driessen et al., 2001; Driessen, 2003; Andersen et al., 2005). Comparative studies confirm that Mediterranean populations hold more alleles and show stronger substructure, whereas Northern Atlantic and Baltic groups exhibit high gene flow and low differentiation (Desplanque et al., 1999; Leys et al., 2014; Richards et al., 2014; Veloso et al., 2021; Bertram et al., 2025a).

Specifically Baltic and North Sea populations exhibit genetic homogeneity and low polymorphism, reflecting founder effects and bottlenecks associated with recent colonization and seed dispersal via ocean currents (Driessen et al., 2001; Driessen, 2003). Danish and Swedish populations show high gene flow and no internal structure, consistent with wind pollination and long-distance dispersal (Andersen et al., 2005; Bertram et al., 2025a). These northern groups remain distinct from Mediterranean and Atlantic populations, reflecting their restricted genetic base and recent origin.

Different dispersal mechanisms also influence population structure. Coastal populations showed strong geographic clustering shaped by marine currents (Leys et al., 2014). Inland ruderal populations exhibit more complex genetic structures due to admixture and human activities, such as soil and plant movement and habitat modification (Letschert, 1993; Leys et al., 2014). These actions introduce and mix genetically distinct individuals, increasing gene flow and hybridization, and disrupting clear geographic genetic patterns. Nuclear genes, spread by both pollen and seeds, enable broader gene flow and genetic mixing across regions. In contrast, mitochondrial genes, dispersed only through seeds, are more restricted in their movement, resulting in stronger spatial genetic structuring (Fénart et al., 2008). For example, along the French Atlantic and Channel coasts, an asymmetric gene flow shaped by marine currents and differences in nuclear versus cytoplasmic dispersal was observed (Fievet et al., 2007). This demonstrates how the mode of gene transmission shapes genetic patterns in wild plant populations.

Within these broader patterns, distinct regional variations introduce additional complexity. Admixture signals, such as clustering between French Atlantic and Corsican individuals, highlight ongoing connectivity despite distance (Richards et al., 2014). Similar dynamics occur in Iberian and Macaronesian systems, where marine currents and isolation create admixture gradients. Northern groups are more differentiated, while southern and insular populations form mixing zones (Veloso et al., 2021).

Environmental heterogeneity also plays a role in genetic differentiation of populations. For instance, the presence of mixed ploidy levels in Portuguese *Beta* taxa (Castro et al., 2013) and the high genetic diversity found in salt marsh populations (Ribeiro et al., 2016) point to a strong adaptive potential linked to diverse habitats. Such habitat heterogeneity creates a mosaic of selective pressures, fostering local adaptation and maintaining genetic variation. Additionally, the pronounced variability observed in Madeira and Porto Santo demonstrates that environmental selection can sometimes override the effects of geographic distance, leading to distinct population characteristics even within relatively small regions (Ascarini et al., 2021).

Overall, geographic distance alone is an unreliable proxy for genetic differentiation. Population structure results from glacial history, dispersal mechanisms, and ecological selection.

## Discussion

Research on sea beet populations has progressed from early morphological studies to increasingly sophisticated genetic analyses. Initial work focused on phenotypic traits to infer diversity and adaptation, revealing substantial variation shaped by geography, environment, and local selection pressures. With the advancement of molecular techniques, research has shifted toward examining genetic diversity and population structure, offering deeper insights into evolutionary processes and genetic diversity (Desplanque et al., 1999; Andersen et al., 2005; Leys et al., 2014; Bertram et al., 2025a).

A key observation is that phenotypic variation in sea beet populations often exceeds genetic differentiation and is strongly influenced by environmental conditions (Ribeiro et al., 2016; Ascarini et al., 2021; Abdelhameed et al., 2024). The species’ outcrossing mating system, along with high pollen and seed dispersal, promotes gene flow and hence genetic mixing among populations. At the same time, the high phenotypic plasticity of *Beta maritima* enables individuals to respond flexibly to strong environmental gradients across their range. This plasticity complicates the interpretation of morphological data, especially when environmental variation is pronounced. As a result, morphological traits may not reliably reflect underlying genetic relationships. While some studies have identified major genes, such as the *B* gene for vernalization requirement, comprehensive integration of phenotypic and genetic data remains limited. Notably, there are successful examples of wild alleles being introgressed into cultivated beet, such as the *Rz2* gene for Rhizomania resistance (Capistrano-Gossmann et al., 2017). However, most research has focused on either morphological or genetic variation in isolation, so the extent to which genetic architecture explains morphological patterns is only partially examined. Agronomic traits, especially disease and pest resistances, have been evaluated only minimally across sea beet populations. This gap, likely also due to the difficulty of evaluating such traits in wild material directly (Bertram et al., 2025b), highlights the need for further studies to systematically assess and characterize this diversity.

Comparative studies have consistently identified two distinct genetic groups among sea beet populations – one from the Atlantic and one from the Mediterranean region, with the latter generally exhibit greater genetic and morphological diversity (Desplanque et al., 1999; Richards et al., 2014). This divide has been further confirmed by sequence analyses of 239 sea beet *ex-situ* accessions from germplasm banks (Sandell et al., 2022; Felkel et al., 2023). This division reflects the influence of both historical events and evolutionary processes on population structure. During the last glacial period, Mediterranean regions acted as refugia and preserved high ancestral diversity. In contrast, post-glacial recolonization toward the north caused genetic bottlenecks and reduced allelic richness in northern populations (Driessen et al., 2001; Driessen, 2003; Andersen et al., 2005).

Nevertheless, this does not mean that northern populations lack valuable alleles for breeding. For example, Capistrano-Gossmann et al. (2017) identified the *Rz2* resistance gene to Rhizomania, which is of major importance for breeding, in a Danish sea beet population. Other studies have also reported unique alleles and polymorphisms in northern Atlantic populations (Andersen et al., 2005; Bertram et al., 2025a). While it remains unclear which of these alleles hold practical value for breeding, the implications are significant. This highlights the importance of *in-situ* conservation, specifically of genetically unique micro-populations, to preserve unique genetic variants. It also underscores the need for broad sampling and comprehensive testing to fully uncover useful genetic diversity across all regions. Genomic tools, including high-density SNP arrays and whole-genome sequencing, can aid with the identification of candidate alleles for introgression and the systematic assessment of genetic resources (Andrello et al., 2017; Felkel et al., 2023).

**Figure 2:**
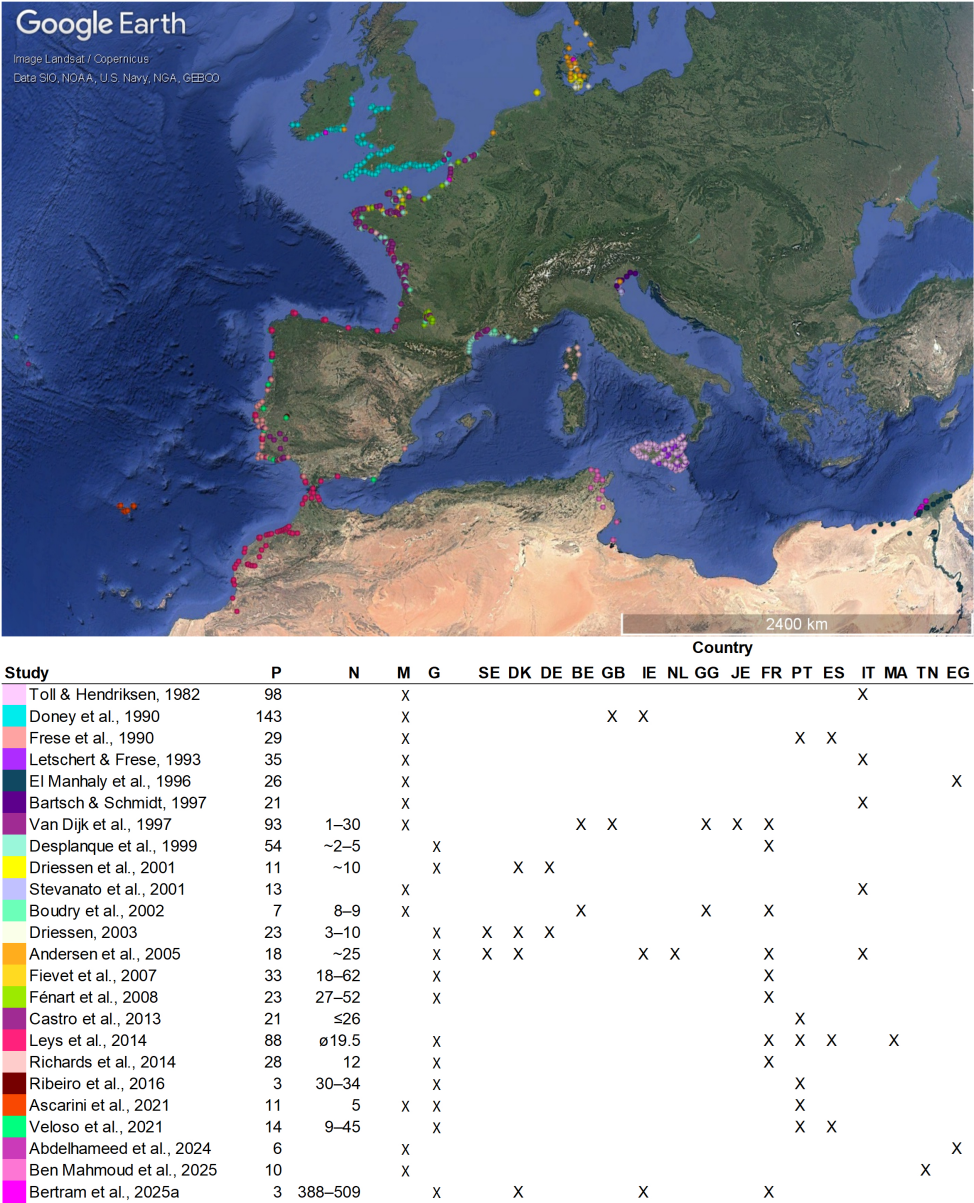
Geographic distribution of *Beta maritima* populations sampled within the studies covered by this literature review. Locations are based either on GPS coordinates provided in the original publications or inferred from maps therein. Populations are color-coded by study. Only *Beta maritima* populations are shown. P = total number of sea beet populations (locations) sampled, N = number of individuals per sampled population. Type of study: M = morphological: G = genetical characterization. Countries from which populations were evaluated with each study are indicated: SE, Sweden; DK, Denmark; DE, Germany; BE, Belgium; IE, Ireland; NL, The Netherlands; GG, Guernsey; JE, Jersey; FR, France; PT, Portugal; ES, Spain; IT, Italy; MA, Morocco; TN, Tunesia; EG, Egypt. The map was generated using GoogleEarthPro.

Mediterranean populations, with their high diversity and high amount of unique alleles, surely represent valuable sources for crop improvement. Especially their ability to thrive even under challenging conditions, such as drought or high salinity, makes them especially valuable for breeding programs aiming to develop more resilient cultivars. However, despite evidence of rich diversity, Mediterranean sea beet populations remain notably underrepresented, especially in genetic studies (**Figure 2**). Notable gaps exist along the southern and eastern coasts of Spain, the Italian coastline and Sardinia, all of Greece, the western coast of Turkey, and other eastern Mediterranean regions. Although genebank accessions sampled from these areas confirm the historical presence of *Beta maritima* (Andrello et al., 2016), many of these populations have not been genetically characterized. Zucchini et al. (2024) also noted discrepancies between the presence of *in-situ* populations and the original collection sites of *ex-situ* accessions. One possible reason for this is the ongoing decline of natural habitats, which threatens the survival of sea beet populations (Doney et al., 1990; Stevanato et al., 2001). However, this does not fully explain the lack of data, as recent studies still report widespread occurrences, for example along the Italian coast (Zucchini et al., 2024). This highlights a significant research gap. Although Mediterranean populations are known to exist and contribute substantial diversity, they remain largely uncharacterized. Future research should prioritize these regions to better capture the full spectrum of sea beet diversity and its breeding potential.

Despite recent progress, the value of many genetic studies on sea beet populations is limited by small sample sizes and a narrow set of genetic markers, restricting insights into the full genetic architecture of these populations (**Figure 2**). While these studies provide useful estimates of heterozygosity and allelic richness in specific regions, they offer only a partial view of overall diversity. The work by Bertram et al. (2025a) represents a major advancement, applying high-density SNP genotyping across large and diverse populations. This approach enables a more comprehensive analysis of genetic variation, population substructure, and mapping potential, setting a new benchmark for future research. However, while genome-wide SNP data represent a significant improvement over single-marker approaches, SNP panels are often developed based on cultivated material and may suffer from ascertainment bias (Andrello et al., 2017), potentially underrepresenting rare or novel alleles in wild populations. To fully capture the genetic diversity in *Beta maritima* populations and detect also structural variants such as insertions and deletions (Felkel et al., 2023), whole-genome sequencing would be preferable. Sequencing technologies are becoming increasingly accessible and are likely to become the method of choice for future diversity studies.

Ultimately, the value of sea beet for crop improvement depends not only on the presence of genetic diversity but also on the ability to identify and utilize alleles conferring desirable traits. A key challenge remains: How to evaluate breeding potential without extensive, resource-intensive testing? First studies have begun to tackle this question. Capistrano-Gossmann et al. (2017) demonstrated an approach to identify resistance genes directly within sea beet populations without time-consuming material development. Building on this, Bertram et al. (2025b) used simulation studies to design suitable development schemes for evaluating even complex traits like yield.

Looking forward, addressing current knowledge gaps through integrated genomic, phenotypic, and environmental research is essential to fully harness the potential of *Beta maritima* for sugar beet improvement. Combining morphological, genomic, climatic, and soil data will provide a more comprehensive understanding. Genome-wide scans and landscape genomics can reveal adaptive variants and clarify the environmental drivers of genetic differentiation. Additionally, systematic phenotyping for stress and disease traits, together with the integration of *ex-situ* and *in-situ* datasets, will help resolve inconsistencies and maximize the utility of wild genetic resources for both breeding and conservation.

In summary, sea beet populations exhibit remarkable morphological and genetic diversity shaped by geography, environment, and dispersal dynamics. The transition from morphological to genetic characterization has greatly enhanced our understanding of wild sea beet diversity. Nevertheless, significant gaps remain, particularly in the underexplored Mediterranean populations and in translating genetic variation into breeding value to unlock the full value of *Beta maritima* populations for resilient and sustainable crop development.
